# Simulation of Waveforms of a Ferrite Inductor with Saturation and Power Losses

**DOI:** 10.3390/ma7031850

**Published:** 2014-03-04

**Authors:** Rosa Ana Salas, Jorge Pleite

**Affiliations:** Electronic Technology Department, Higher Polytechnic School, Carlos III University of Madrid, Avda. de la Universidad, 30, 28911 Leganés (Madrid), Spain; E-Mail: pleite@ing.uc3m.es

**Keywords:** ferrite cores, Finite Element Analysis (FEA), power losses, nonlinear inductors

## Abstract

We propose a model of an equivalent electrical circuit specifically designed for a ferrite indAuctor excited by a sinusoidal waveform. The purpose of this model is its use in a circuit simulator. We calculate the model parameters by means of Finite Elements in 2D which leads to significant computational advantages over the 3D model. We carry out the validation for a toroidal ferrite inductor by comparing the experimental results and computed ones. We consider the saturation and power losses in the core. In addition, we have tested the model for the case of square waveform in order to generalize the results. We find excellent agreement between the experimental data and the results obtained by numerical calculations.

## 1. Introduction

MnZn soft ferrites are widely used in the field of power electronics due to their low power losses, high saturation induction, and low cost [1–6]. These ferrites show nonlinear effects in the core, such as hysteresis and saturation, which should be included in a model in order to carry out precise transient simulations [7–21]. Due to the great variety of shapes, sizes and number of turns in the winding, it is difficult to develop a model that is both simple and precise. Models and linear simulations of these ferrites are found in the literature [22–27]. In spite of these studies there is a lack of models that incorporate simultaneously different geometries and sizes and a wide range of working frequencies and regions, including linear, intermediate and saturation regions.

**Figure 1.**
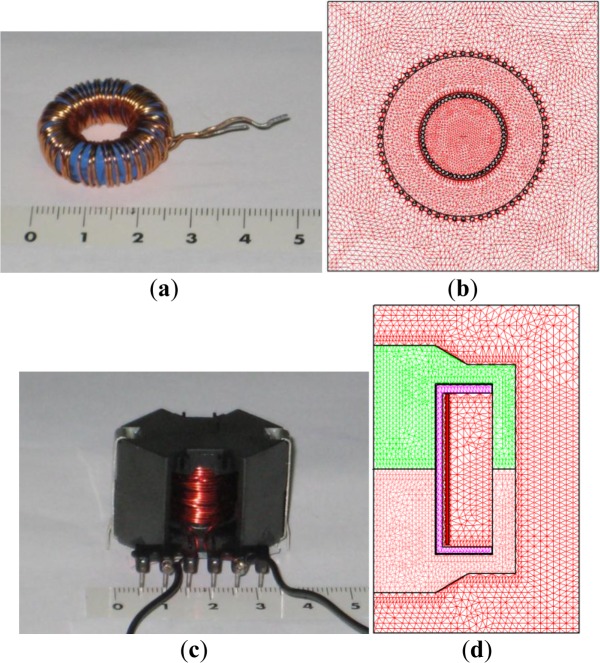
(**a**) Real inductor with a toroidal ferrite core; (**b**) triangular mesh generated by 2D simulations; (**c**) real inductor with an RM (Rectangular Modulus) ferrite core; (**d**) triangular mesh generated by 2D simulations.

In this paper we present an electrical model of a ferrite inductor specifically valid for simulating the voltage and current waveforms of an inductor with a circuit simulator. We obtain the model parameters by 2D Finite Element Analysis. As an example, we show the results for a toroidal-shaped inductor (Figure 1a) excited by sinusoidal voltage waveforms at different frequencies. We present the results for all working regions of the ferrite (linear, intermediate and saturation) and consider the effect of power losses in the core. The main advantage of our methodology is a reduction of the computational cost and the inclusion of the effect of frequency in the simulations. In order to generalize the model for other waveforms and different core shapes, we will present the results obtained for the case of an RM inductor (Figure 1c) excited by a square waveform at two different frequencies. These waveforms were generated by a buck DC-DC power converter working at 40 and 100 kHz.

The paper is organized as follows. The results are presented and compared with experimental measurements in Section 2. In Section 3 we present the model of the equivalent electrical circuit of the inductor. We also present the methodology that we propose to obtain the waveforms through the circuit simulator. In addition, we show the procedure used to obtain the experimental measurements. These measurements were necessary to validate the model and as input parameters to carry out the Finite Element simulations. Finally, in Section 4 we summarize our results and offer some conclusions.

## 2. Results

First, we will present the results of the sinusoidal waveform and afterwards the corresponding results for the square waveform. We have applied our procedure to an inductor with a toroidal ferrite core that consists of a TN23/14/7 ferrite core and 60 turns of copper wire. In Figure 1a we show the built inductor. In order to verify the model, we excite the tested inductor with a sinusoidal waveform at two different frequencies. A low frequency of 50 Hz (quasistatic condition) and 20 kHz (working frequency of this ferrite). To obtain these experimental data we follow the measuring method described in Section 3.2.2. For this purpose we increase the amplitude of the voltage waveform from 0 V until the saturation of the material is reached. We carried out the measurements at 50 Hz in a laboratory at Carlos III University of Madrid. We used two variable transformers to excite the inductor, a 1 Ω resistance to measure the current through the inductor, and a Tektronix TDS360 digital oscilloscope (Tektronix, Inc., Beaverton, OR, USA) to measure the waveforms. For the measurements at 20 kHz we used an ultrasonic power amplifier constructed at CSIC (Spanish National Research Council) to excite the inductor. This amplifier is capable of supplying 500 W and an output voltage of 160 V*_rms_*. In addition, we also use an Agilent 33120A function generator to provide the sinusoidal waveform, a Tektronix TDS360 digital oscilloscope to measure the waveforms and a 1 Ω resistance to measure the current through the inductor.

In order to take the experimental measurements necessary to validate the *L-I* curve computed by the 2D FEA simulations, another identical copper coil was wound around the first one to build a transformer. In this case we use the methodology described in Section 3.2.1.

We carry out the measurements of the *B-H* and *Φ-I* curves at the Polytechnic University in Madrid. We use a Magnet-Physik electronic fluxmeter EF 4 (MAGNET-PHYSIK Dr. Steingroever GmbH, Köln, Germany) to measure the magnetic flux, a Xantrex XFR 2.8 kW DC power supply (Xantrex, Elkhart, IN, USA) to excite the inductor, and a Fluke 8010A digital multimeter (Fluke Corporation, Everett, WA, USA) to measure the current through the inductor.

We carry out the 2D simulations using the Ansoft Maxwell field simulator’s magnetostatic solver in the *x*-*y* plane to compute the *L-I* curve and the transient solver to compute the *R*−*I_rms_* curve. In Figure 1b we show the corresponding 2D equivalent design made for the 2D FEA simulations. We chose *D* (*x*,*y*)∈ [−30, 30] [−20, 20] (unit are millimeters) as a computational domain and τ = 1% as a fixed percent error. In order to run the simulations we consider both the leakage inductance of the ferrite and the parasitic capacity to have no effect. For the simulations we have taken the values of the Steinmetz coefficients for each frequency obtained by experimental measurements. For the simulations at 50 Hz using the methodology described in Section 3.2.2 we get the values of the Steinmetz coefficients as *C_m_* = 5.2, α = 1, β = 1.2 and for 20 kHz we get these values as *C_m_* = 8.33, α = 1.26, β = 1.86. We have also computed the value of the DC resistance of the winding according to *R*_Ω_ = *ℓ*/σ*S* ≈ 0.0936Ω being σ = 5.8×10^7^ Sm^−1^ the copper’s conductivity, *S* the cross-sectional area of the copper wire used and *ℓ* its length. Note that the winding resistance is more important at low working frequencies.

We have carried out the validation of the procedure based on the *L-I* and *R*−*I_rms_* curves computed by 2D FEA simulations and the waveforms obtained by the circuit simulator PSIM (Powersim Inc., Rockville, MD, USA), comparing for each case the experimental and simulated results.

**Figure 2.**
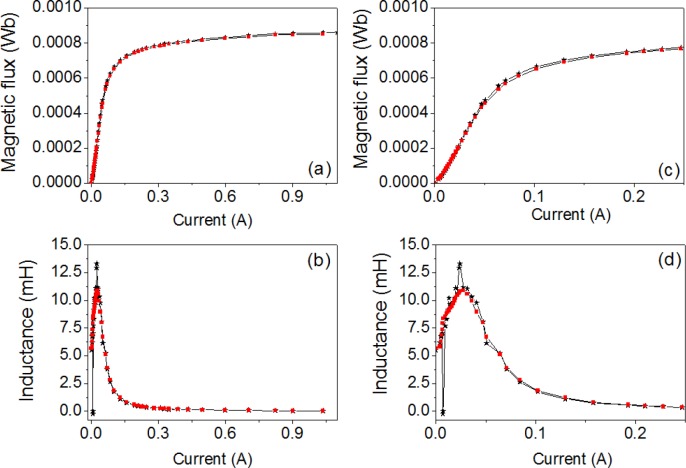
(**a**) Magnetic flux curves; (**b**) *L-I* curves; (**c**) magnetic flux curves in the range of [0 A, 0.25 A]; (**d**) *L-I* curves in the range of [0 A, 0.25 A]. Experiment (– _*_ –) and 2D simulations (–▪–).

**Figure 3.**
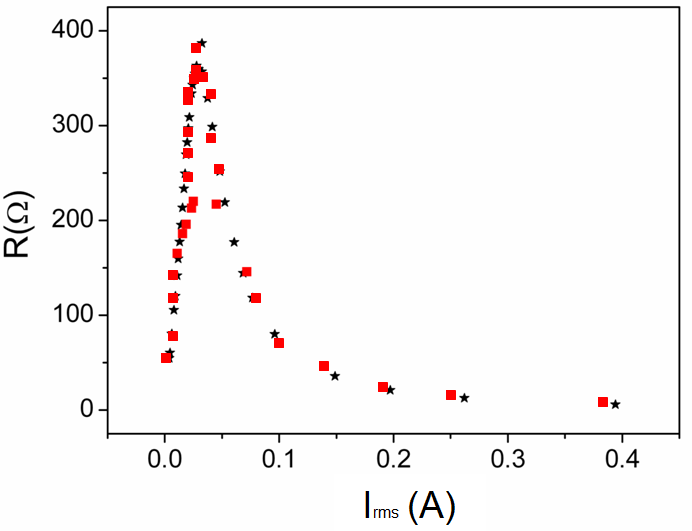
Results at 20 kHz. *R*−*I_rms_* curve. Experiment (– _*_ –) and 2D simulation (–▪–).

In Figure 2 we plot both the Φ-*I* and *L-I* curves showing good agreement between the results. Both the magnetic flux and the inductance exhibit a nonlinear relation to the DC excitation current applied to the core. The saturation values tend to 0.0008 Wb, obtaining similar values in 2D and experiment. The *L-I* curve reaches a maximum and tends to a very small final value with increasing *I*. This maximum corresponds to the inflexion point of the magnetic flux curve. As an example in Figure 3 we show the resistance curve at 20 kHz, where we see that the resistance increases when the *I_rms_* current increases. There is good agreement between the experimental and computed resistance values.

**Figure 4.**
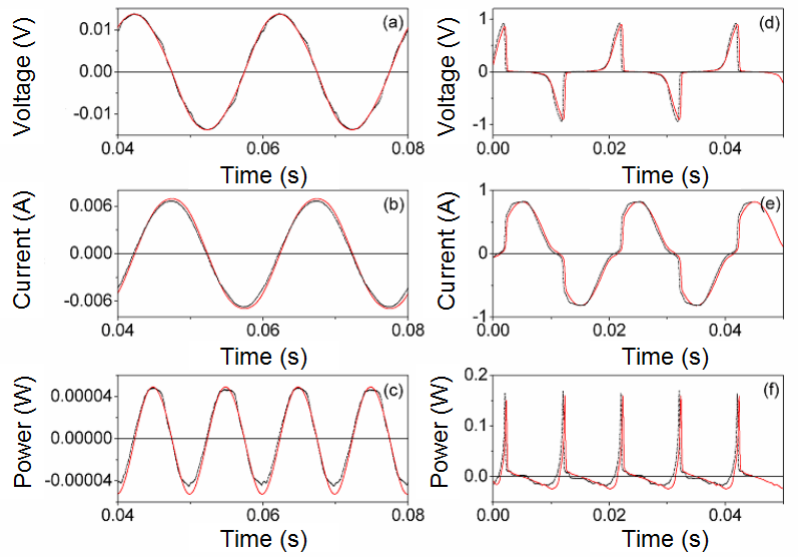
Experimental (-·-·-) and simulated waveforms at 50 Hz **(─)**. (**a**–**c**) *V_p_* ≈ 0.0135 V; (**d**–**f**) *V_p_* ≈ 0.92 V and time step τ = 2 × 10^−7^ s.

**Figure 5.**
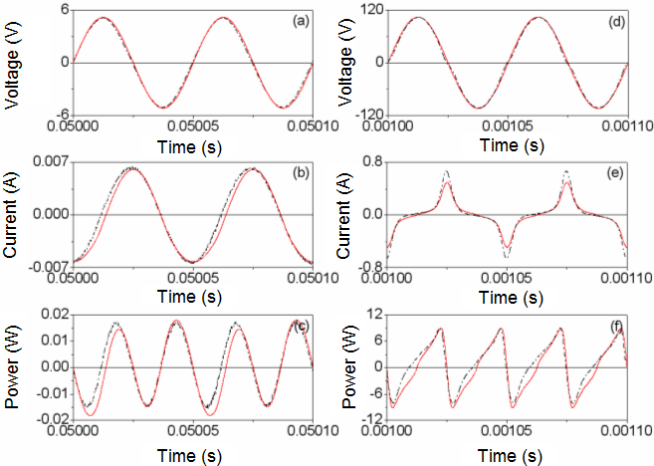
Experimental (-·-·-) and simulated waveforms (**─**) at 20 kHz. (**a**–**c**) *V_p_* ≈ 5.24 V and time step τ = 10^−6^ s. (**d**–**f**) *V_p_* ≈ 104.75 V and time step τ = 2 × 10^−8^ s.

Next, in Figures 4 and 5 we show the results of the PSIM waveforms at frequencies of 50 Hz and 20 kHz for two different amplitudes *V_P_* of the sinusoidal voltage applied to the inductor. Figures 4a–c and 5a–c correspond to a value of *V_P_* in the linear region and Figures 4d–f and 5d–f are the results for a value of *V_P_* where the core is saturated. As can be seen, there is good agreement between the results. The saturation effect is included in the model as the current waveform is curved. In order to verify that the effect of power losses is reproduced in our model we compute the average power in a cycle, defined as (3). In Table 1 we show the experimental $\bar{P_{\exp}}$ and simulated $\bar{P_{mod}^{R}}$ power values computed at 50 Hz and 20 kHz and for different amplitude values of the sinusoidal voltage (*V_p_*). As we can see, our model is capable of reproducing the effect of power losses. The powers $\bar{P_{mod}^{R}}$, although small, increase with frequency and to a lesser extent with *V_p_* as typically observed experimentally. This is due to the fact that an increase of the voltage leads to an increase of the current through the inductor. As a consequence, both *Φ* and *B* increase, as well as the size of the generated hysteresis loop and the power losses in the core. When we do not consider power losses in the core, the value of the average power $\bar{P_{mod}}$ is zero, as theoretically expected.

As commented before, in order to generalize the proposed model of the equivalent electrical circuit, we will present representative results obtained for the case of a nonsinusoidal excitation. These waveforms are typically used in power electronic applications. To do this, we excited the tested inductor by a square waveform provided by a buck DC-DC power converter. We built this converter in order to be capable of supplying the range of voltage, current and frequency needed to reach saturation of the ferrite core. For this purpose, we constructed a 10-turn RM14/I inductor made of 3F3 material with a 1 mm diameter copper wire winding. In Figure 1c we show the constructed inductor.

We carried out the experimental measurements and the construction of the converter in a laboratory at Carlos III University of Madrid. In order to construct the converter we used a 450 μF capacitor, an IRF530A power MOSFET transistor (Fairchild Semiconductor Corporation, San Jose, CA, USA), an output filter, and a 3.1 Ω load resistance. The output filter consisted of the tested inductor and a 450 μF capacitor. In order to take the necessary experimental voltage and current waveforms at different frequencies we used an Agilent HP 6012B DC power supply to provide the DC input voltage, a Tektronix DM2510 digital multimeter to measure the DC input and output voltages (*V_i_* and *V*_0_ respectively). In addition, we used an Agilent 33120A function generator to change the duty cycle of the MOSFET transistor and a Tektronix TDS5104 digital oscilloscope to measure the voltage and current waveforms of the inductor. In order to take the necessary experimental measurements of the inductance, we follow the methodology previously described in Section 3.2.1. The only difference is that for the two-winding transformer we used an RM14/I ferrite core. In order to obtain the experimental *R*−*I_rms_* curve, as well as the necessary experimental data of average power and Steinmetz coefficients to carry out the simulations, we follow the methodology described in Section 3.2.2 using square waveforms. We carried out the 2D simulations using the magnetostatic solver in the *r-z* plane to compute the *L-I* curve and the transient solver to compute the *R*−*I_rms_* curve. For the 2D transient simulations the values of the Steinmetz coefficients we used are *C_m_* = 8, α = 1.4, β = 1.5. We computed these values from the experimental waveforms at 100 kHz following the methodology described in Section 3.2.2.

**Figure 6.**
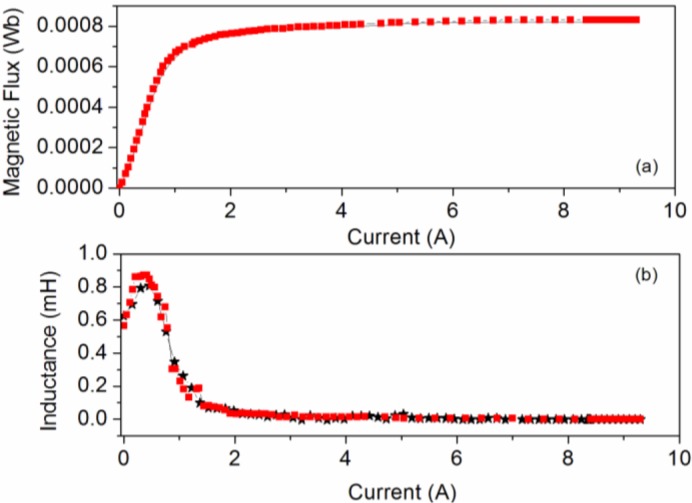
(**a**) Magnetic flux curves. (**b**) *L-I* curves. Experiment (– _*_ –) and 2D simulations (–▪–).

**Figure 7.**
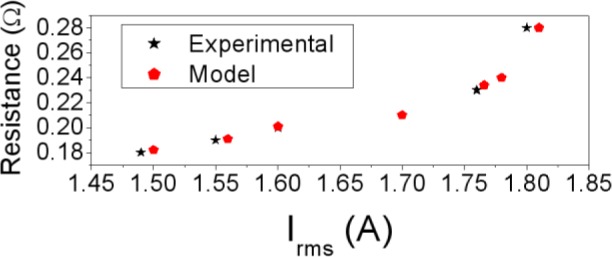
Results at 100 kHz. *R*−*I_rms_* curve. Experiment (– _*_ –) and 2D simulation (–▪–).

In Figure 1d we show the 2D equivalent inductor model designed for the 2D FEA simulations and the generated mesh. For this purpose, we chose *D*: (*r*,*z*) ∈ [0, 25] × [−20, 20] (unit are millimeters) as a computational domain and τ = 1% as a fixed percent error. In Figure 6 we show the *L-I* curve obtained by experiment and 2D simulations. As can be seen, the results are in good agreement as happens in the case of the sinusoidal excitation using the toroidal ferrite core. In both cases the curves show the same behavior. The inductance has a maximum and tends to a very small final value when the current increases. In Figure 7 we show the corresponding experimental and simulated results of the *R*−*I_rms_* curve at 100 kHz. In both cases the experimental and simulated results are in good agreement. As the current increases, the power increases, as happens experimentally.

**Figure 8.**
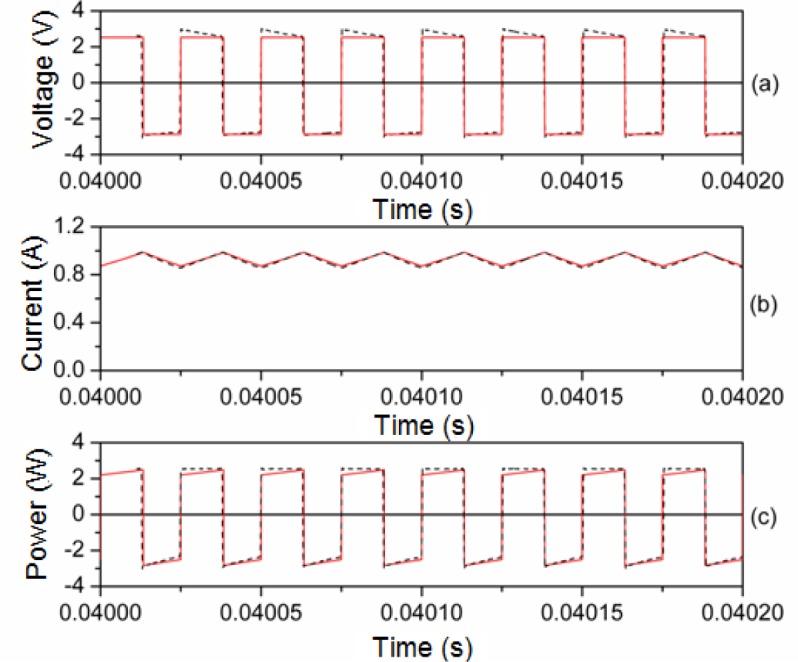
Experimental (-·-·-) and simulated (**─**) waveforms at 40 kHz. *V_in_* ≈ 5.4 V and time step τ = 10^−8^ s.

**Figure 9.**
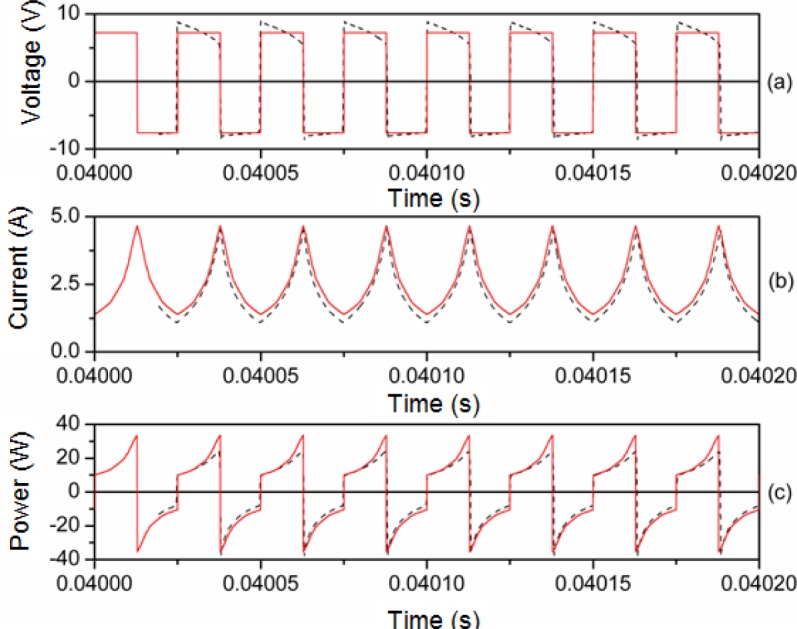
Experimental (-·-·-) and simulated (**─**) waveforms at 40 kHz. *V_in_* ≈ 12 V and time step τ = 10^−8^ s.

**Figure 10.**
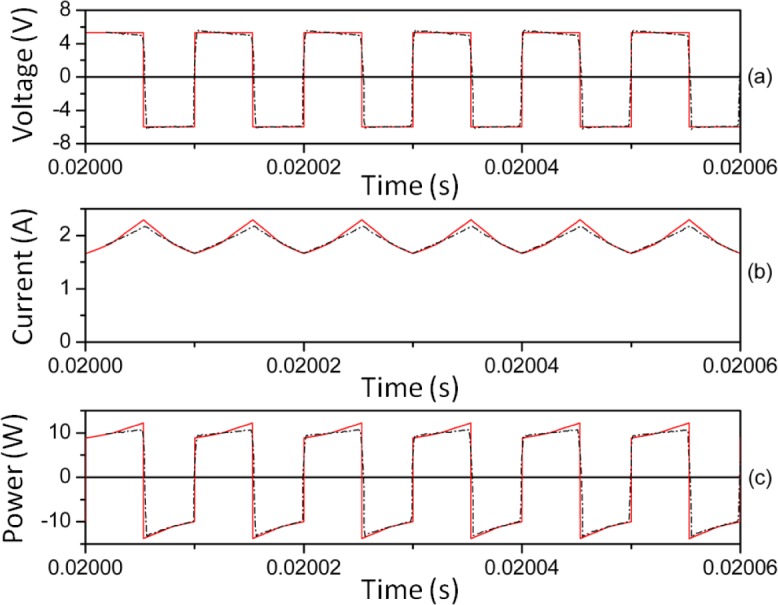
Experimental (-·-·-) and simulated (**─**) waveforms at 100 kHz. *V_in_* ≈ 11.68 V and time step τ = 10^−8^ s.

**Figure 11.**
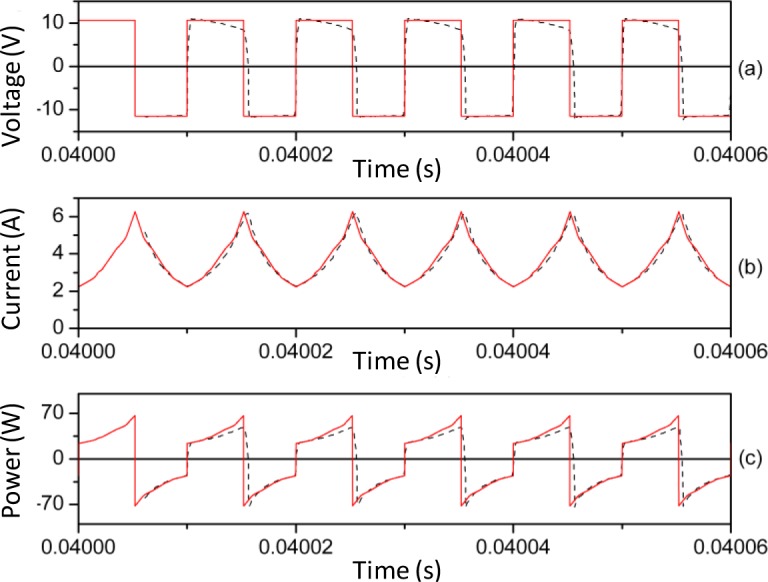
Experimental (-·-·-) and simulated (**─**) waveforms at 100 kHz. *V_in_* ≈ 22.63 V and time step τ = 10^−8^ s.

In Figures 8 and 9 we plot representative waveforms obtained by experiment and by simulation through PSIM at 40 kHz and a duty cycle of 0.5 approximately. In Figures 10 and 11 we show the waveforms at 100 kHz. For both frequencies we show the results for two different DC input voltages *V_in_* of the power converter. We show the results for the linear region in Figures 8 (*V_in_* ≈ 5.4 V, 40 kHz) and 10 (*V_in_* ≈ 11.68 V, 100 kHz). The results for the saturation region are represented in Figures 9 (*V_in_* ≈ 12 V, 40 kHz) and 11 (*V_in_* ≈ 22.63 V, 100 kHz) [28].

As can be seen, as happens in the case of the sinusoidal excitation, there is good agreement between the experimental and simulated results. The effect of saturation is reproduced since the current waveform is curved. This curve would not exist if the model was linear. The triangular shape of the current waveforms shown in Figures 8b and 10b is typical for power converters working in the linear region.

In order to verify that the effect of power losses are also reproduced in our model, in Table 2 we present the experimental and computed powers for three values of *V_in_* (linear, intermediate and saturation regions) and two frequencies (40 kHz and 100 kHz). As can be seen and as happens in the case of the sinusoidal excitation, the computed powers $\bar{P_{mod}^{R}}$, although small, increase with frequency and to a lesser extent with *V_in_* as typically observed experimentally.

## 3. Materials and Methods

### 3.1. Obtaining the Waveforms from the Electronic Circuit Simulator

**Figure 12.**
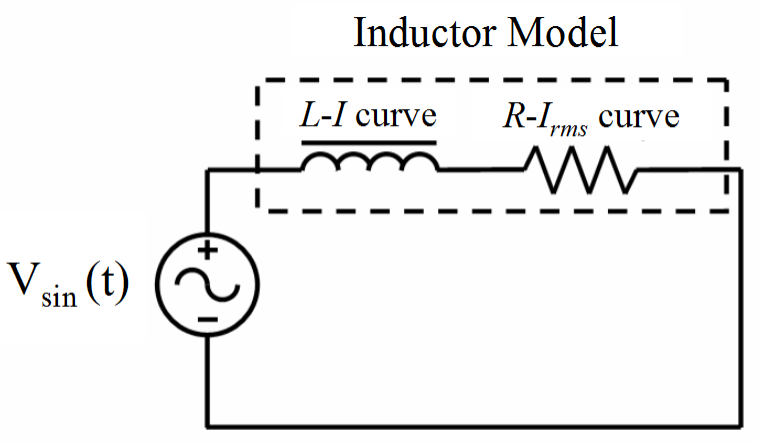
Diagram representing the used inductor model.

The main objective of this section is to describe the methodology that we propose for obtaining the inductor waveforms from a circuit simulator. We studied the case of an inductor excited by a sinusoidal voltage waveform. For this purpose we consider the inductor model to be an equivalent electronic circuit consisting of an inductance *L* and power loss resistance *R* connected in series (Figure 12). In our model both *L* and *R* are nonlinear.

*L* is a function of the DC current *I* and *R* is a function of the rms current *I_rms_* and frequency. To obtain the values of the *L* and *R* curves we use a software based on Finite Element Analysis. Typical 3D simulations require high computational cost and may have convergence problems so we propose a simulation procedure in 2D. This procedure converges and substantially reduces the computational cost. To obtain the *L-I* curve we use a magnetostatic solver, and to obtain the *R*−*I_rms_* curve we use a transient solver. The reason for choosing a magnetostatic solver to calculate *L* is that we have been able to verify experimentally that *L* varies very little with frequency.

**Figure 13.**
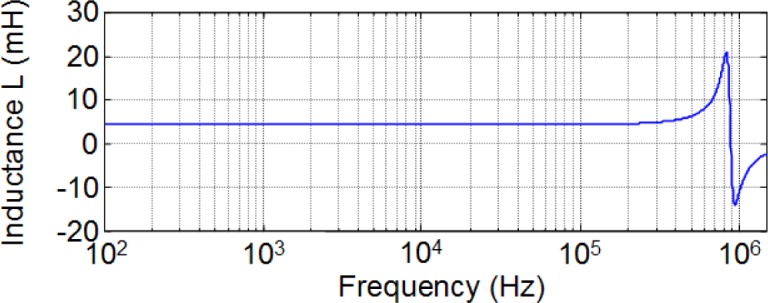
Inductance measurements as a function of frequency for a 28-turn RM14/I ferrite inductor.

**Figure 14.**
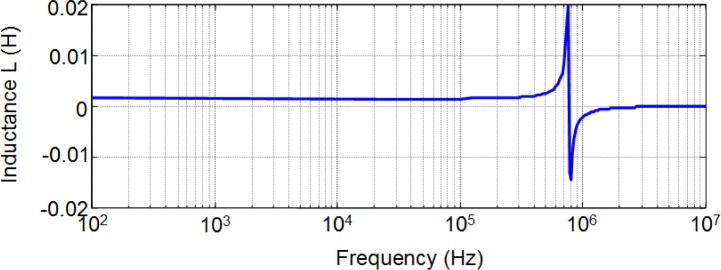
Inductance measurements as a function of frequency for a 28-turn E47/20/16 ferrite inductor.

To investigate the frequency dependence of *L* in a wide range of operation frequencies up to the order of Megahertz we have carried out experimental measurements in the form of frequency sweep using a precision impedance analyzer. This includes the typical operation frequencies used for many switched mode power supply applications (higher than 100 kHz). We carried out the measurements in a laboratory at Carlos III University of Madrid. To do this we used a Hewlett Packard 4194A precision Impedance/Gain-Phase Analyzer (Agilent Technologies, Santa Clara, CA, USA). In Figure 13 we show the results for a 28-turn RM14/I ferrite inductor and in Figure 14 we show the results for a 28-turn E47/20/16. In both cases the sweep covers frequencies from 100 Hz to over 1 MHz. As can be seen, the inductance *L* is approximately constant and independent of frequency. The resonant frequency for the RM inductor is about 832 kHz while in the case of the E inductor it is about 768 kHz. The observed variations of *L* correspond to the resonant frequency.

The modeling procedures of *L* and *R* involve three steps: *premodeling*, *simulation* and *postmodeling*. These procedures will be detailed later in this paper. We have developed a computer software based on Matlab, Simulink and the circuit simulator PSIM, where we insert the values of the *L-I* and *R*−*I_rms_* curves. We obtain the voltage and current waveforms from PSIM and from these data derive the power waveform. Finally, we compare the results with experimental measurements.

In order to calculate the *L-I* curve, at the *premodeling step* we measure the magnetic properties of the ferrite (*B-H* curve) and design the 2D equivalent inductor model. This model includes background, core geometry, winding and coil-former if present. In the case of a toroidal core we use a cross-section of the real inductor as shown in Figure 1b. Next, we assign the magnetic properties of the materials. These magnetic properties are the relative permeability of the background and copper winding, the conductivity of the copper winding and the *B-H* curve of the ferrite material. In addition, we assign the boundary conditions and the values of the DC excitation current *I*. We also assign the adaptive parameters to generate the mesh. These parameters are the percent refinement per pass, number of requested passes and percent error τ. We choose adaptive analysis to request that the finite element mesh be adaptively refined in areas with the highest error such as the corners of the core.

At the *simulation step* we introduce the premodeling data in the Maxwell software and run the program.

At the *postmodeling step* we obtain as an output of the software the spatial distribution of the magnetic fields **B** and **H**. Regarding the notation related to magnetic fields, **B** and **H** (in bold) represent the magnetic field vectors. Then, from these data we compute the *Φ-I* curve and from this we derive the *L-I* curve by differentiation.

In order to calculate the *R*−*I_rms_* curve, we follow a similar procedure but we carry out a 2D simulation in time domain. At the *premodeling step* we use as input to the simulation program, apart from the *B-H* curve, the values of the Steinmetz coefficients of the ferrite to be analyzed. The physical meaning of these coefficients is described in the literature [29–36]. The coefficients account for the power losses. In order to measure these data we follow the methodology described in Section 3.2. From here on, the design of the 2D model and assignment of the magnetic properties of the inductor materials are the same as the previous procedure to obtain the *L-I* curve, the only exception being that the excitation applied to the inductor is a sinusoidal voltage instead of a DC current. Due to this fact, we need to assign values of the voltage amplitude, frequency and details of the windings such as initial current, resistance *R*_Ω_, leakage inductance, capacitance and number of turns.

At the *simulating step* we obtain the voltage, current and power waveforms and the instantaneous spatial distribution values of the magnetic fields **B** and **H**.

At the *postmodeling step* we compute $\bar{P}$ (average value of the power) and *I_rms_* (rms values of the current waveform). The *R* value is calculated as $R=\bar{P}/I_{rms}^{2}$. By repeating the simulation at different excitation voltage values, we obtain the $\bar{P}-I_{rms}$ *curve* and from this derive the *R*−*I_rms_* *curve*.

This calculation can be repeated for each working frequency needed. We have validated the procedure by comparing these results with experimental measurements.

In the case of a square excitation, we follow the same methodology. The only difference is that as we use an RM ferrite core, we use its corresponding 2D equivalent model for the simulations [37] (Figure 1c,d).

We have applied our methodology to a MnZn soft ferrite core made of 3F3 material from the manufacturer Ferroxcube (Hispano Ferritas-Ferroxcube, Guadalajara, Spain). Its main application area is power transformers and inductors as well as general purpose transformers. This material has an initial permeability μ*_i_* at 25 °C = 2000 ± 20%, a saturation flux density *B_sat_* = 440 mT at 25 °C and H = 1200 A/m, Curie Temperature T_C_ = 200 °C and a DC resistivity ρ ≈ 2 Ωm.

### 3.2. Experimental Measurements

We took two different types of measurements to analyze the nonlinear behavior of the ferrite cores to obtain the input parameters of the 2D FEA simulations and to validate the proposed procedure and simulations by comparison with the experimental results. These two different types of measurements are measurements under a variable DC current as well as under variable voltage and frequency.

#### 3.2.1. Measurements under Variable DC Current

The objective of the measurements is to obtain the *B-H* curve that characterizes the core material of the ferrite from the linear to the saturation regions. This curve is an input parameter in the simulation program. We derive the *B-H* curve from the *Φ-I* curve. For this we build a two-winding transformer with the same number of turns in the primary (*N_p_*) and secondary (*N_s_*) coils. For the transformer we use a toroidal ferrite core with the same material as the inductor to be studied. We apply a variable DC current *I*, generated from a DC power supply, to the primary coil. We measure the magnetic flux *Φ* in the secondary coil with the Magnet-Physik electronic fluxmeter (Köln, Germany) from the linear to the saturation regions. With these values we compute *B* and *H* as follows:

$$B\approx \frac{1}{N_{s}A_{e}}\int v\;dt=\frac{\Phi }{N_{s}A_{e}}$$ (1)

$$H\approx \frac{N_{p}I}{\ell_{e}}$$ (2)

where *A_e_* is the effective cross-sectional area of the core, and *^ℓ^_e_* is the effective magnetic path length. These values are defined in the Ferroxcube catalog for each geometry.

#### 3.2.2. Measurements under Variable Voltage and Frequency

The objectives of these measurements are to obtain the Steinmetz coefficients, to validate the output voltage and the current waveforms obtained from the proposed procedure to estimate *R*, as well as validating the output voltage and current waveforms from the circuit simulator.

For this purpose, we measure the voltage *v*(*t*) and current *i*(*t*) waveforms by exciting the inductor with a sinusoidal waveform in order to obtain the $\bar{P}-I_{rms}$ curve from which we derive the *R*−*I_rms_* curve and the ${\bar{P}}_{v}-B_{p}$ curve from which we derive the Steinmetz coefficients. $\bar{P}$ and ${\bar{P}}_{v}$ are respectively the average value of the power and the average value of the power per unit volume of the core. We compute $\bar{P}$ as:

$$\bar{P}=\frac{1}{T}\int_{0}^{T}v(t)\times i(t)dt$$ (3)

where *T* is the time period of the waveform. We then compute *R* using the equation. $R=\bar{P}\text{/}I_{rms}^{2}$.

In order to obtain the values of the Steinmetz coefficients α, β, *C_m_* we fit the experimental data ${\bar{P}}_{v}-B_{p}$ to the Steinmetz equation,

$${\bar{P}}_{v}=C_{m}f^{\alpha }B_{p}^{\beta }$$ (4)

In this equation *B_p_* is the maximum value of the magnetic field *B*(*t*) defined as;

$$B(t)=\frac{1}{N\;A_{e}}\int v\left(t\right)dt$$ (5)

where *N* is the number of turns of the inductor to be studied.

In order to obtain the necessary experimental data to validate the model for a square waveform, we follow the same methodology, the only difference being that we use a square waveform to excite the inductor.

## 4. Conclusions

In this paper we have presented a model of an equivalent electrical circuit specifically valid for an inductor with a toroidal ferrite core. The purpose of the model is its use in a circuit simulator. The main advantages are the reduction of the computational cost in an electronic circuit simulator and improved convergence. In particular, we have applied the model to an inductor with a toroidal-shaped ferrite core with 60 turns excited by a sinusoidal voltage at two different frequencies and for different excitation levels of the ferrite (linear, intermediate and saturation). We have developed a computer software based on PSIM, Matlab and Simulink, where we have implemented the equivalent electrical circuit of the inductor consisting of *L* and *R* in series considering two cases: with and without power losses in the core. We have used a Finite Element software to obtain the values of *L* and *R* curves from Finite Element Analysis in 2D. This software allows us to obtain the output voltage and current waveforms of the inductor. We have shown two representative examples, one for 50 Hz (low frequency) and one for 20 kHz (working frequency of ferrite) where we have compared the resulting voltage, current and power waveforms obtained from the PSIM circuit simulator with the equivalent experimental waveforms. We have also compared the simulated and experimental results of *L* and *R*. As we have been able to verify, our model reproduces the effects of saturation and power losses, showing good agreement between the experimental and computed results. Both the waveforms and the *L* and *R* curves follow the same tendency as the experimental results. We have also been able to verify that the power values, although small, increase with frequency and to a lesser extent with the amplitude of the sinusoidal waveforms as typically observed experimentally. In order to generalize the model we have also tested the same electrical model for the case of a square waveform using a different shape of the core than the toroidal one. To do this, we included a 10-turn RM14/I ferrite inductor in a buck DC-DC power converter. We then compared the waveforms obtained by experiment and by simulation at 40 kHz and 100 kHz. We also compared the simulated and experimental results of *L* and *R*. We could again verify that our model reproduces the effects of saturation and power losses, showing good agreement between the experimental and computed results. We observed that the power increases with the DC input voltage of the converter and the voltage and current waveforms follow the same tendency as the experimental results. The power losses, although small, increase when the frequency increases.

This shows that our model can be applied to different frequencies and waveforms from the linear to the saturation regions.

As future work a possible extension of this work would be the development of a more complete electrical equivalent model of the inductor. This model would consist of a capacitance *C* in parallel to *L* and *R* in series. This would account for the parasitic capacitance of the windings.

## Figures and Tables

**Table 1.** Experimental and modeled average powers for two different values of the amplitude of the voltage waveforms (*V_p_*).

Frequency (kHz)	*V_p_* (V)	$\bar{P_{\exp}}\;(\text{W})$	$\bar{P_{mod}^{R}}\;\left(\text{W}\right)$
0.05	0.013	2.8 × 10^−6^	2.6 × 10^−6^
	0.92	0.0019	0.0017

20	5.24	0.00085	0.00083
	104.75	0.85	0.84

**Table 2.** Experimental and modeled average powers for three different values of input voltage of the converter (*V_in_*).

Frequency	*V_in_* (*V*)	$\bar{P_{\exp}}\;(\text{W})$	$\bar{P_{mod}^{R}}\;\left(\text{W}\right)$
40 kHz	5.40	0.075	0.08
	6.98	0.12	0.13
	12.00	0.17	0.16

100 kHz	11.68	0.31	0.32
	19.34	0.74	0.73
	22.63	1.085	1.1
